# Effects of Vitamin D, Melatonin, and Omega-3 Fatty Acids on Periodontal Health: A Narrative Review

**DOI:** 10.3390/dj13040178

**Published:** 2025-04-20

**Authors:** Dora Dragičević Tomičić, Nikolina Lešić, Ivana Škrlec, Larissa Steigmann, Kristina Tseneva, Martina Čalušić Šarac, Tin Crnić, Igor Tomičić, Željka Perić Kačarević, Marija Čandrlić

**Affiliations:** 1 Department of Dental Medicine, Faculty of Dental Medicine and Health Osijek, J.J. Strossmayer University of Osijek, Crkvena 21, 31000 Osijek, Croatia; 2 Department of Biophysics, Biology and Chemistry, Faculty of Dental Medicine and Health Osijek, J.J. Strossmayer University of Osijek, Crkvena 21, 31000 Osijek, Croatia; 3 Department of Oral Medicine, Infection and Immunity, Division of Periodontology, Harvard School of Dental Medicine, Boston, MA 02115, USA; 4 Botiss Biomaterials AG, Ullsteinstrasse 108, 12109 Berlin, Germany; 5 Health Center of Osijek-Baranja County, Park Kralja Petra Krešimira IV 6, 31000 Osijek, Croatia; 6 Department of Periodontology and Operative Dentistry, University of Mainz, Augustusplatz 2, 55131 Mainz, Germany; 7 Department of Anatomy, Histology, Embriology, Pathology Anatomy and Pathology Histology, Faculty of Dental Medicine and Health Osijek, J.J. Strossmayer University of Osijek, 31000 Osijek, Croatia; 8 Department of Integrative Dental Medicine, Faculty of Dental Medicine and Health Osijek, J.J. Strossmayer University of Osijek, 31000 Osijek, Croatia

**Keywords:** periodontal diseases, vitamin D, fatty acids, omega-3, melatonin

## Abstract

Periodontitis is a chronic inflammatory disease characterized by the destruction of tooth-supporting structures, influenced by immune system dysregulation, oxidative stress, and imbalances in bone metabolism. Given its multifactorial pathogenesis, bioactive compounds such as vitamin D, melatonin, and omega-3 fatty acids have emerged as potential adjuncts to periodontal therapy due to their immunomodulatory, anti-inflammatory, and antioxidative properties. This narrative review explores the role of these three supplements in periodontal health, their potential in synergistic effects, and existing research gaps, providing a foundation for future studies on their clinical applications. Vitamin D is essential for calcium homeostasis, bone remodeling, and immune function. It modulates both innate and adaptive immune responses, enhancing antimicrobial peptide production and reducing inflammatory cytokine expression. Omega-3 fatty acids reduce the production of pro-inflammatory eicosanoids while promoting the synthesis of pro-resolving lipid mediators, contributing to bone preservation and immune balance. Melatonin, known for its antioxidant and osteogenic properties, supports bone remodeling by stimulating osteoblast proliferation and inhibiting osteoclast activity, while also regulating circadian rhythms, which may influence oral health. Although these bioactive compounds show promising effects in preclinical and clinical studies, significant knowledge gaps remain regarding optimal dosages, long-term efficacy, combined use, and standardized treatment protocols. Further clinical trials are necessary to elucidate their therapeutic value in periodontal disease management, especially those focused on their potential synergistic mechanisms. Understanding their synergistic mechanisms may open new avenues for adjunctive strategies in periodontal therapy.

## 1. Introduction

Periodontitis is a chronic inflammatory disease that gradually destroys the tissues supporting the teeth, resulting in alveolar bone resorption and, if left untreated, eventual tooth loss [1,2]. While bacterial dysbiosis is a crucial in its initiation, the progression of periodontitis is largely impacted by the host’s immune response. Intrinsic and extrinsic factors contribute to disease severity, including immune system dysregulation, oxidative stress, systemic conditions such as diabetes and HIV (Human Immunodeficiency Virus), lifestyle factors like smoking, and nutritional deficiencies [3,4,5]. A key issue of periodontitis is the disruption of bone homeostasis, where chronic inflammation promotes excessive bone resorption and disturbs regenerative processes [6]. Given the complexity of its pathogenesis, increasing attention has been directed toward external modifiable factors, such as nutrition, which can influence periodontal health and disease progression [7]. Bioactive substances have emerged as potential adjuncts to conventional periodontal therapy, as they can modulate the immune and inflammatory response and promote bone regeneration, thereby supporting periodontal health [2,8].

In this context, nutrition plays a fundamental role not only in maintaining overall systemic health but also in influencing periodontal health. Nutrients are categorized into macronutrients, proteins, carbohydrates, and fats, which provide the structural and energy basis of the diet, and micronutrients, which are required in smaller amounts, but are essential for various physiological functions [9,10]. A well-balanced diet supplies critical nutrients that support immune function, regulate inflammation, and contribute to the maintenance of healthy oral tissues. Recent research highlights the role of bioactive compounds in modulating inflammation, oxidative stress, and bone metabolism, key processes in periodontal disease progression [11,12]. Among these, vitamin D, omega-3 fatty acids, and melatonin stand out due to their distinct, yet complementary mechanisms, which directly target the pathophysiological pathways of periodontal destruction and regeneration [9].

The selection of these three supplements is supported by clinical and preclinical evidence demonstrating their synergistic potential in addressing the inflammatory, oxidative, and bone-related pathways of periodontitis. Woelber et al. [13] demonstrated that a diet rich in omega-3 fatty acids, vitamin D, and antioxidants significantly reduced gingival inflammation and periodontal pocket depths, reinforcing the clinical relevance of these nutrients in periodontal therapy. Furthermore, a study by Beyer et al. [14] examined the interplay between marine omega-3 fatty acids, vitamin D, and periodontal health in rheumatoid arthritis patients, revealing that a higher omega-3 index was associated with lower periodontal probing depth and improved systemic inflammation markers. The beneficial effects of vitamin D, omega-3 fatty acids, and melatonin in conditions such as sarcopenic obesity and rheumatoid arthritis demonstrate their broader systemic relevance. Since periodontitis shares common inflammatory mechanisms with these disorders, these supplements may offer therapeutic value in periodontal disease management [15]. Therefore, this narrative review focuses on vitamin D, omega-3 fatty acids, and melatonin due to their well-documented but under-synthesized roles in periodontitis and their potential for synergistic benefits. By consolidating existing evidence, this review aims to provide a broader understanding of their biological interactions in periodontal therapy, while also identifying gaps for future research regarding optimal dosage, administration, and long-term effects in clinical practice.

## 2. Literature Search

This narrative review was conducted to explore the potential roles of vitamin D, omega-3 fatty acids, and melatonin in periodontal health, with a focus on their immunomodulatory, anti-inflammatory, antioxidative, and osteogenic properties. A literature search was finalized in January 2025 using the PubMed and Google Scholar databases. The search included preclinical animal studies and clinical human research that examined the effects of these supplements on periodontal health. To ensure a comprehensive literature search, a combination of relevant keywords related to vitamin D, melatonin, omega-3 fatty acids, and periodontal health was applied, without restrictions on the publication year. Studies were evaluated based on their relevance to dental medicine, particularly those investigating oral supplementation and local applications of these bioactive compounds. The review process prioritized studies that provided clinical or experimental evidence of the effects of vitamin D, omega-3 fatty acids, and melatonin in the context of periodontal disease. Articles were included if they assessed the role of these supplements in periodontal inflammation or as adjuncts to periodontal therapy. Only studies published in English were considered to maintain clarity and accuracy in data interpretation. Research that primarily examined systemic diseases, orthopedic applications, or in vitro cell cultures was excluded to keep a focus of analysis on periodontal health. Through qualitative synthesis, this review aims to discuss on the relevance of these bioactive compounds in periodontal therapy, while also identifying key knowledge gaps and future research directions.

## 3. Vitamin D and Periodontal Health

### 3.1. Vitamin D, Bone Metabolism, and Immunomodulation

**Figure 1:**
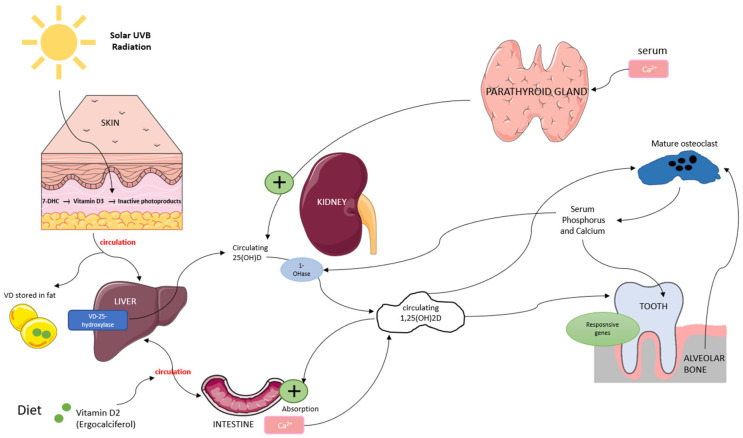
Overview of vitamin D metabolism and its role in bone remodeling. Vitamin D is synthesized in the skin or absorbed from the diet, converted in the liver to 25(OH)D, and then activated in the kidneys to 1,25(OH)_2_D, which regulates calcium-phosphate balance and supports osteoblast and osteoclast function.

Vitamin D is a fat-soluble secosteroid obtained through sun exposure, dietary intake, and supplementation. It plays a crucial role in bone metabolism by regulating calcium and phosphorus homeostasis, which is essential for bone mineralization and remodeling. Vitamin D exists in two primary forms: vitamin D3 (synthesized in the skin through ultraviolet B (UVB) exposure) and vitamin D2 (derived from dietary sources). In the skin, UVB radiation converts 7-dehydrocholesterol (7-DHC) into vitamin D3, while vitamin D2 from food is absorbed into the bloodstream. Both forms bind to vitamin D-binding protein (VDBP) and are transported to the liver, where they are converted by 25-hydroxylase into 25-hydroxyvitamin D (25(OH)D), the main biomarker for vitamin D status [16,17]. In the kidneys, 25(OH)D undergoes further hydroxylation by 1α-hydroxylase to form 1,25-dihydroxyvitamin D (1,25(OH)_2_D), its biologically active form. This activation process is regulated by serum calcium and phosphorus levels through a negative feedback mechanism involving parathyroid hormone (PTH). In bone metabolism, 1,25(OH)_2_D promotes osteoblast function while stimulating osteoclast differentiation, facilitating controlled bone resorption and the release of calcium and phosphorus necessary for remodeling [16,18,19] (Figure 1).

In the periodontium, vitamin D receptors (VDRs) are widely expressed, and the active form, 1,25-dihydroxyvitamin D (1,25(OH)_2_D), plays a crucial role in bone metabolism and immune regulation. It regulates osteoblast and osteoclast activity, maintaining the balance between bone formation and resorption. Studies have shown that vitamin D stimulates the expression of osteogenic markers such as runt-related transcription factor 2 (Runx2), osterix (Osx), and alkaline phosphatase (ALP), all essential for osteoblast differentiation and function [20,21,22]. Beyond its role in bone metabolism, vitamin D exerts immunomodulatory effects by binding to VDRs expressed in various immune cells, including dendritic cells, monocytes, and B cells [23,24]. Through VDR activation, it modulates antigen-presenting cell (APC) function, enhances macrophage anti-inflammatory activity, and reduces the proliferation of pro-inflammatory T-helper (Th1 and Th17) cells while promoting the differentiation of regulatory T cells (Tregs) [20,25]. Vitamin D also plays a key role in controlling inflammatory cytokines. It downregulates pro-inflammatory mediators, including tumor necrosis factor alpha (TNF-α), interleukin 1β (IL-1β), and interleukin 6 (IL-6) while upregulating interleukin 10 (IL-10), an anti-inflammatory cytokine essential for immune homeostasis [26,27].

### 3.2. Effects of Vitamin D on Periodontal Health

Periodontitis is a chronic inflammatory disease marked by alveolar bone resorption, largely driven by an imbalanced immune response to microbial biofilms. The role of vitamin D in immune regulation is particularly relevant in this context. Studies consistently show the association between vitamin D levels and periodontitis [28,29]. Patients with periodontitis have been reported to exhibit significantly lower vitamin D levels compared to healthy individuals [30,31,32,33,34,35,36]. Gong et al. [37] found that vitamin D deficiency impairs osteoblastogenesis, leading to alveolar bone loss, even when calcium and phosphorus levels are sufficient. Furthermore, Alshouibi et al. [38] analyzed data from 562 patients and found that vitamin D supplementation correlated with improved periodontal outcomes, including reduced alveolar bone loss, pocket depth, and clinical attachment loss.

The effects of vitamin D supplementation on periodontal health have demonstrated heterogeneous outcomes, with some studies indicating potential benefits. Garcia et al. [39] conducted a one-year cohort study evaluating the effects of systemic vitamin D and calcium supplementation on chronic periodontitis patients. The study observed significant improvements in clinical parameters at six months, although the effects diminished after 12 months, suggesting a potential short-term benefit. Similarly, Miley et al. [40] found that combined vitamin D and calcium supplementation resulted in borderline-significant improvements (*p* = 0.08) in periodontal health, indicating a possible adjunctive role for these nutrients. A pilot study by Meghil et al. [41] explored the influence of 12-week vitamin D supplementation on systemic and salivary inflammatory markers in periodontitis patients. The results suggested immune regulation mediated by vitamin D, which may provide localized benefits in periodontal disease treatment, particularly in vitamin D-deficient patients. Studies on vitamin D as an adjunct to non-surgical periodontal therapy (NSPT) suggest potential but inconsistent benefits. Gao et al. [42] found modest short-term effects, while Perić et al. [43] and Lei et al. [44] observed greater improvements in probing pocket depth (PPD), clinical attachment level (CAL), and bleeding index (BI) with higher weekly doses over six months. A meta-analysis by Liang et al. [45] confirmed significant CAL improvements with supplementation, but limited effects on other clinical parameters. Additionally, a systematic review and meta-analysis by Machado et al. [46] included sixteen studies and found that serum 25(OH)D levels were significantly lower in patients with chronic periodontitis compared to healthy individuals. However, the authors noted a lack of consistent evidence regarding the effects of vitamin D supplementation after NSPT, particularly due to a low number of intervention studies. These findings reinforce the need for further high-quality clinical trials to clarify the impact of vitamin D supplementation in periodontal therapy.

Beyond systemic supplementation, Kirkwood et al. [47] demonstrated that topical vitamin D reduced inflammation, shifted the oral microbiome toward a healthier composition, and enhanced pro-resolving mediator production in a mouse model. These findings suggest vitamin D—both systemic and topical—may complement periodontal therapy, particularly in deficient patients, though further research is needed to establish optimal dosing and long-term efficacy.

### 3.3. Knowledge Gaps and Future Directions for Vitamin D in Periodontology

Many studies have reported borderline-significant or short-term effects, indicating that vitamin D alone may not be sufficient as a standalone therapy, but could serve as an adjunct to periodontal therapy [12,13,24,25,29]. Several key areas require further investigation, as follows. (1) Defining optimal vitamin D levels for periodontal health: While some studies suggest benefits in vitamin D-deficient individuals, the threshold for therapeutic efficacy in periodontology is still unclear. (2) Longitudinal studies: Many clinical trials report short-term improvements, but longitudinal studies are needed to assess the sustained effects of vitamin D on periodontal stability. (3) Combined therapies: Future research should explore the synergistic effects of vitamin D with other bioactive compounds, particularly omega-3 fatty acids and melatonin, given their complementary anti-inflammatory, antioxidant, and osteogenic properties. (4) Local vs. systemic administration: Some studies suggest that local vitamin D application may offer targeted benefits compared to systemic supplementation, warranting further clinical trials. While vitamin D supplementation has demonstrated potential adjunctive benefits in periodontal therapy, more robust, high-quality RCTs with standardized protocols are necessary to confirm its clinical efficacy and practical applications.

## 4. Omega-3 Fatty Acids and Periodontal Health

### 4.1. Anti-Inflammatory Mechanisms of Omega-3 Fatty Acids in Periodontal Disease

**Figure 2:**
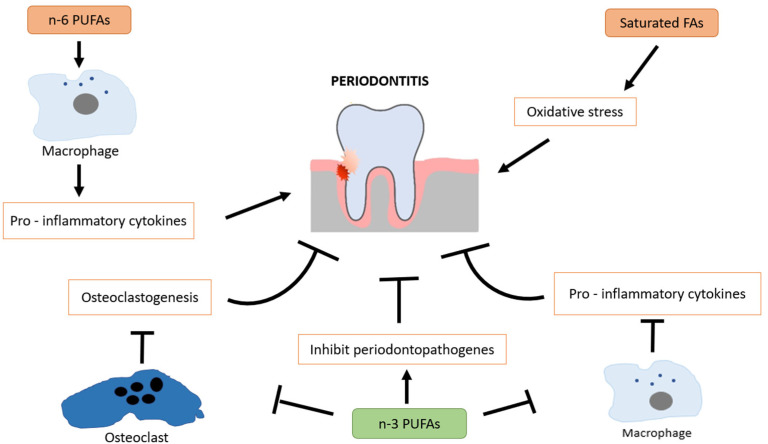
Protective role of omega-3 fatty acids in periodontal health through modulation of inflammation, immune balance, and oxidative stress.

Omega-3 fatty acids are polyunsaturated fatty acids (PUFAs) found primarily in fish oil, flaxseeds, and walnuts, with eicosapentaenoic acid (EPA) and docosahexaenoic acid (DHA) being the most biologically active forms. These essential fatty acids are well known for their systemic anti-inflammatory and immunomodulatory effects, which may contribute to slowing the progression of periodontal disease [48]. A key mechanism by which omega-3 fatty acids exert their effects is through the regulation of eicosanoids, including prostaglandin E_2_ (PGE_2_), thromboxanes, and leukotrienes, which are involved in inflammatory responses. Omega-3 fatty acids are converted into specialized pro-resolving mediators (SPMs) such as resolvins, protectins, and maresins, which actively promote resolution of inflammation by reducing neutrophil infiltration, inhibiting pro-inflammatory cytokines, and increasing monocyte recruitment [49,50]. Omega-3 fatty acids play a protective role in periodontitis by modulating inflammatory bone resorption and supporting alveolar bone preservation. Their effects are inversely correlated with periodontal tissue breakdown. As potent anti-inflammatory agents, omega-3 fatty acids promote the acquisition of a pro-resolving macrophage phenotype, contribute to immune homeostasis, and suppress the production of pro-inflammatory cytokines. In addition to their well-established anti-inflammatory properties, omega-3 polyunsaturated fatty acids (n-3 PUFAs) have been shown to exert antimicrobial activity by inhibiting the growth of periodontopathogenic bacteria [51,52]. This adds another layer to their therapeutic relevance in periodontal disease. In contrast, omega-6 PUFAs (n-6 PUFAs) tend to promote a pro-inflammatory phenotype in macrophages, contributing to tissue destruction when imbalanced with n-3 intake [53]. Saturated fatty acids (SFAs) are also implicated in periodontal pathology, as they are associated with increased oxidative stress and inflammatory signaling, which may exacerbate periodontal tissue breakdown [54] (Figure 2).

A recent preclinical animal study by Gad et al. [55] investigated the impact of systemic administration of omega-3 PUFAs in a rabbit model, reporting that omega-3 supplementation significantly reduced osteoclastic activity. This osteoclastic inhibitory effect suggests a protective role in preserving alveolar bone structure, which may be relevant in periodontitis. Van Dyke et al. [56] explored the role of pro-resolving nanomedicines containing a lipoxin analogue (bLXA4) in promoting bone regeneration in periodontitis. Their results demonstrated that treatment with bLXA4 reduced inflammatory cell infiltration, increased new bone formation, and enhanced periodontal tissue regeneration. Similarly, Vardar et al. [57] examined the therapeutic versus prophylactic administration of omega-3 fatty acids in endotoxin-induced periodontitis in rats. They found that local injection of omega-3 significantly reduced inflammatory mediators, including prostaglandin E_2_ (PGE₂), prostaglandin F₂ alpha (PGF₂α), leukotriene B_4_ (LTB_4_), and platelet-activating factor (PAF). Interestingly, prophylactic omega-3 administration combined with therapeutic use resulted in the most favorable reduction in inflammation, restoring tissue condition closer to that of healthy controls. These results shed the light on the potential dual role of omega-3 fatty acids in both preventing and treating periodontal inflammation.

### 4.2. Clinical Studies on Omega-3 Supplementation in Periodontal Therapy

Human clinical studies have provided mixed, but generally promising results regarding the role of omega-3 supplementation as an adjunct to periodontal therapy. Kujur et al. [58] conducted an RCT evaluating omega-3 supplementation combined with NSPT in patients with chronic periodontitis. Their findings demonstrated significant improvements in clinical parameters, including reduced probing pocket depth, improved clinical attachment level, and lower gingival inflammation scores compared to conventional therapy alone. Similarly, Stańdo et al. [59] investigated the effects of high-dose omega-3 supplementation in patients with stage III and IV periodontitis, reporting beneficial effects on periodontal inflammation and clinical healing. In a specific RCT conducted on postmenopausal women, Elgendy et al. [48] evaluated the impact of omega-3 supplementation on periodontal parameters and systemic antioxidant status. Their findings revealed that omega-3 supplementation led to reduced periodontal inflammation and improved systemic antioxidant enzyme activity. Additionally, Maybodi et al. [60] reported that omega-3 supplementation, when compared to soybean oil, significantly improved periodontal parameters, highlighting its potential as a dietary adjunct in periodontal therapy. However, some studies have found limited or no significant improvements. Martinez et al. [61] found that omega-3 supplementation had no effect on the clinical outcomes of periodontal treatment when combined with conventional therapy, suggesting that its benefits may be highly dependent on patient-specific factors. Keskiner et al. [62] also reported that low-dose omega-3 supplementation reduced salivary TNF-α levels, but had no significant effect on clinical parameters, indicating that while omega-3 may show systemic anti-inflammatory effects, these may not always translate directly into clinical periodontal improvements. Conversely, studies assessing combination therapies have shown more consistent positive outcomes. Naqvi et al. [63] found that omega-3 supplementation combined with low-dose aspirin significantly improved periodontal outcomes, suggesting it as a less expensive and safer method for the prevention and treatment of periodontitis. Similarly, Elkhouli et al. [64] reported that systemic administration of omega-3 plus low-dose aspirin resulted in reduced gingival inflammation, improved attachment levels, and modulation of cytokine expression.

### 4.3. Limitations, Risks, and Future Research Needs for Omega-3 in Periodontology

Despite the promising evidence supporting omega-3 fatty acids in periodontal health, several limitations and considerations should be noted. First, variability in study protocols, dosages, and duration of supplementation makes it challenging to establish standardized clinical recommendations. Second, while omega-3 supplementation appears to modulate inflammatory responses, its effects on bone metabolism and tissue regeneration require further validation through long-term trials. Moreover, high-dose omega-3 supplementation can carry potential risks, including gastrointestinal discomfort, increased bleeding tendencies (particularly in patients taking anticoagulants), and oxidative stress due to lipid peroxidation [65,66]. These safety concerns confirm the importance of individualized patient assessments before recommending omega-3 supplementation as part of periodontal therapy. Future research should focus on standardizing omega-3 dosages and treatment duration in periodontal therapy, investigating the long-term impact of omega-3 on bone metabolism and periodontal stability, exploring combination therapies for better outcomes, and assessing patient-specific responses to omega-3 supplementation to determine who may benefit most from this intervention.

## 5. Melatonin and Periodontal Health

### 5.1. Biological Functions of Melatonin in Periodontal Health

Melatonin, a neurohormone primarily secreted by the pineal gland, is widely known for its role in regulating circadian rhythms and the sleep–wake cycle. However, growing evidence suggests that melatonin also has important antioxidant, immunomodulatory, and anti-inflammatory properties, making it a potential therapeutic agent in periodontal health [67]. Some studies suggest that melatonin may contribute to reducing the severity of oral mucositis after radiotherapy, highlighting its broader relevance in oral health beyond periodontal disease [68]. One of the key mechanisms by which melatonin influences periodontal health is through its antioxidant capacity, which neutralizes oxidative stress in the oral cavity. Oxidative stress plays a major role in the pathogenesis of periodontitis, as it contributes to tissue breakdown and alveolar bone resorption [69]. The presence of melatonin receptors in the gingival tissues enables melatonin to exert direct effects on periodontal tissues by modulating pro-inflammatory cytokine levels and influencing osteoclastogenesis and osteogenesis [70].

### 5.2. Mechanisms of Melatonin in Periodontal Therapy

**Figure 3:**
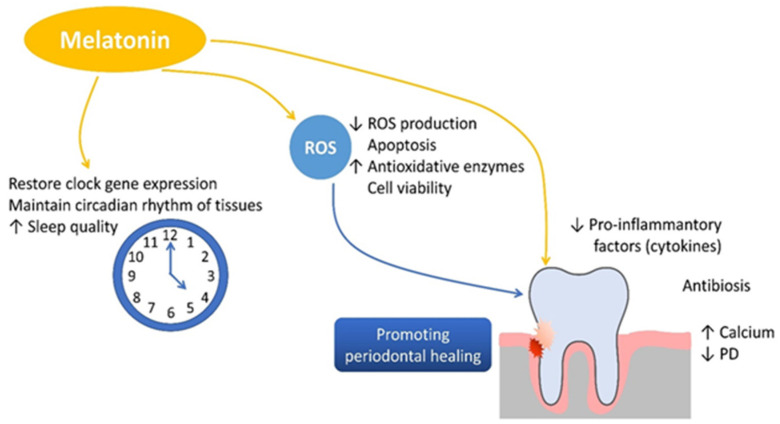
The role of melatonin in promoting bone formation, reducing inflammation, and supporting periodontal health.

In periodontitis, the imbalance between pro-inflammatory and anti-inflammatory mediators leads to excessive activation of osteoclasts and suppression of osteoblast function. Melatonin plays a protective role in this process by downregulating pro-inflammatory cytokines such as tumor necrosis factor alpha (TNF-α), interleukin 1 beta (IL-1β), interleukin 12 (IL-12), interferon gamma (IFN-γ), and interleukin 6 (IL-6) while simultaneously inhibiting matrix metalloproteinases (MMPs), reducing tissue degradation. Additionally, melatonin stimulates the secretion of the anti-inflammatory cytokine interleukin 10 (IL-10) [70,71,72]. In alveolar bone metabolism, melatonin inhibits bone resorption by reducing receptor activator of nuclear factor kappa B ligand (RANKL) expression, thereby preventing osteoclastogenesis. On the other hand, melatonin stimulates osteoblast differentiation by enhancing the expression of runt-related transcription factor 2 (RUNX2) and promoting the synthesis of bone morphogenetic proteins (BMPs), osteocalcin, and osteoprotegerin [70,73,74]. This dual mechanism facilitates new bone formation, accelerates mineralization, and preserves alveolar bone density, all of which are crucial for maintaining periodontal integrity [75,76]. Melatonin is important in bone formation and preservation by stimulating the proliferation and osteogenic differentiation of mesenchymal stem cells, thereby accelerating cartilage and bone formation. It also inhibits osteoclastogenesis by downregulating receptor activator of nuclear factor kappa B ligand (RANKL) expression, thereby preventing osteoclast activation and bone resorption. Additionally, melatonin reduces oxidative stress by neutralizing reactive oxygen species (ROS), contributing to cellular homeostasis. A a key regulator of circadian rhythms, melatonin supports bone tissue maintenance and systemic physiological balance, further reinforcing its potential therapeutic benefits in periodontal health (Figure 3).

Arabacı et al. [77] conducted an experimental study in rats with induced periodontitis, showing that systemic melatonin administration significantly reduced alveolar bone resorption and promoted periodontal tissue regeneration. These findings support melatonin’s potential as a therapeutic agent in periodontal diseases characterized by excessive bone loss. Several human clinical studies have also explored the benefits of melatonin supplementation as an adjunct to periodontal therapy. El-Sharkawy et al. [78] conducted an RCT evaluating dietary melatonin supplementation (10 mg per day) in patients with generalized chronic periodontitis and primary insomnia. Their results demonstrated significant improvements in clinical attachment levels (CAL), probing depth (PD) reduction, and decreased salivary TNF-α levels, demonstrating that melatonin supplementation may enhance periodontal healing, particularly in individuals with sleep disturbances. Additionally, Gonde et al. [79] investigated the effects of locally administered 1% melatonin gel in patients with intrabony defects. Their findings showed that melatonin gel significantly improved both clinical and radiographic outcomes compared to NSPT alone, indicating that local drug delivery systems may improve melatonin’s therapeutic potential. Similar results were observed in Tinto et al. [80], where oral melatonin supplementation (1 mg per day for 30 days) after NSPT led to superior periodontal healing in stage III periodontitis patients compared to NSPT alone. However, not all studies support melatonin’s efficacy in periodontal therapy. Konečná et al. [81] found that melatonin did not significantly improve periodontal outcomes, though the authors acknowledged that the short study duration, method of application, and dosage selection may have influenced the results.

### 5.3. Considerations, Risks, and Future Directions for Melatonin Use in Periodontology

Although melatonin appears to have promising applications in periodontal therapy, certain considerations must be borne in mind. Variability in study design, dosage, and duration of supplementation makes it difficult to establish standardized treatment protocols. Additionally, the route of administration (systemic vs. local) and patient-specific factors may influence melatonin’s effectiveness in periodontal treatment. Regarding safety concerns, melatonin is generally considered well tolerated, but prolonged or high-dose use may disrupt circadian rhythms, leading to sleep disturbances or hormonal imbalances [82]. These potential risks emphasize the importance of individualized treatment approaches and further research to determine optimal dosing regimens for periodontal applications. Future research should focus on: long-term clinical trials evaluating the sustained effects of melatonin on periodontal health, comparative studies investigating different administration methods (oral vs. local application), combination therapies, and determining patient-specific factors that may influence responsiveness to melatonin supplementation. Despite some inconsistencies in study outcomes, melatonin remains a promising candidate for periodontal therapy, particularly due to its antioxidant, anti-inflammatory, and bone-regenerative properties. Well-structured future trials will be essential to establish its precise role and therapeutic potential in periodontology.

## 6. Conclusions

This narrative review explored the potential roles of vitamin D, omega-3 fatty acids, and melatonin in periodontal health, embracing their immunomodulatory, anti-inflammatory, antioxidative, and osteogenic properties. Although clinical and preclinical studies support the role of these bioactive compounds in periodontal therapy, gaps remain regarding optimal dosages, long-term efficacy, and patient-specific responses.

Although strong evidence associates vitamin D deficiency with the severity of periodontitis, further longitudinal studies are required to evaluate its full impact as an adjunct to periodontal therapy. Omega-3 fatty acids show potential in reducing periodontal inflammation, but variations in dosage, study design, and supplementation duration create challenges in forming clinical recommendations. Similarly for melatonin, inconsistent findings suggest that route of administration, dosage, and study duration limit the clarity of evidence of effectiveness of melatonin in periodontal health. While some studies demonstrate significant clinical improvements, others report minimal effects, emphasizing the need for standardized application protocols and longer follow-up periods. Therefore, the development and adoption of unified research protocols, including consistent dosages, administration routes, and outcome measures would allow comparability of findings across studies. This in turn would improve reproducibility, support better clinical translation, and enable the formulation of evidence-based guidelines for the adjunctive and synergistic use of these bioactive compounds in periodontal therapy.

The choice to conduct a narrative review was justified by the diversity and heterogeneity of the available literature on the roles of vitamin D, omega-3 fatty acids, and melatonin in periodontal health. The studies identified varied significantly in terms of design (e.g., in vivo vs. in vitro, animal vs. human), sample size, supplementation protocols, dosage, route and timing of administration, and outcome measures. These methodological differences made it challenging to apply a strict systematic or meta-analytic framework. A narrative review approach was therefore more suitable for a synthesis and interpretation of diverse findings, allowing for a more comprehensive and flexible exploration of the topic. This method enabled us to critically integrate preclinical and clinical data, identify key biological mechanisms, and highlight important knowledge gaps that may inform future research and clinical practice.

The evidence synthesized in this narrative review suggests that vitamin D, omega-3 fatty acids, and melatonin may show synergistic effects in periodontal therapy by targeting distinct, yet complementary biological pathways. Vitamin D plays a crucial role in immunomodulation and bone mineralization, omega-3 fatty acids reduce inflammatory eicosanoids and promote pro-resolving mediators, while melatonin contributes through its antioxidant activity, inhibition of osteoclastogenesis, and promotion of osteoblastic function. Together, these compounds may interact to improve periodontal tissue repair, reduce inflammatory burden, and support alveolar bone preservation more effectively than when used individually. Therefore, future studies should explore this potential by examining combined supplementation protocols, identifying optimal dosage and timing, and clarifying patient-specific factors that influence responsiveness to such adjunctive therapies.

## Data Availability

No new data were created or analyzed in this study.
